# Compromised NK Cell-Mediated Antibody-Dependent Cellular Cytotoxicity in Chronic SIV/SHIV Infection

**DOI:** 10.1371/journal.pone.0056309

**Published:** 2013-02-12

**Authors:** Xuan He, Dan Li, Zhenwu Luo, Hua Liang, Hong Peng, Yangyang Zhao, Nidan Wang, Donghua Liu, Chuan Qin, Qiang Wei, Huimin Yan, Yiming Shao

**Affiliations:** 1 Wuhan Institute of Virology, Chinese Academy of Sciences, Wuhan, China; 2 State Key Laboratory for Infectious Disease Prevention and Control, National Center for AIDS/STD Control and Prevention, Chinese Center for Disease Control and Prevention, Beijing, China; 3 Institute of Laboratory Animal Science, Chinese Academy of Medical Sciences & Peking Union Medical College, Beijing, China; University of Sydney, Australia

## Abstract

Increasing evidence indicates that antibody-dependent cellular cytotoxicity (ADCC) contributes to the control of HIV/SIV infection. However, little is known about the ADCC function of natural killer (NK) cells in non-human primate model. Here we demonstrated that ADCC function of NK cells was significantly compromised in chronic SIV/SHIV infection, correlating closely with the expression of FcγRIIIa receptor (CD16) on NK cells. CD32, another class of IgG Fc receptors, was identified on NK cells with higher expression in the infected macaques and the blockade of CD32 impacted the ability of NK cells to respond to antibody-coated target cells. The inhibition of matrix metalloproteases (MMPs), a group of enzymes normally involved in tissue/receptor remodeling, could restore NK cell-mediated ADCC with increased CD16 expression on macaque NK cells. These data offer a clearer understanding of NK cell-mediated ADCC in rhesus macaques, which will allow us to evaluate the ADCC repertoire arising from preclinical vaccination studies in non-human primates and inform us in the future design of effective HIV vaccination strategies.

## Introduction

Antibody-dependent cellular cytotoxicity (ADCC) is an important bridge between innate and adaptive immunity. Increasing evidence shows a protective role of ADCC in the control of HIV-1 infection [1], [2], [3]. The possibility that non-neutralizing antibodies may mediate protection through ADCC has been seen in assays of HIV candidate vaccines in the non-human primate model [4], [5]. In advance of neutralizing antibody response, systemic non-neutralizing antibodies appeared early during acute infection in both HIV-infected individuals and SIV/SHIV-infected rhesus macaques [6], [7], [8], which implies a greater chance that non-neutralizing antibodies participate in the ADCC response. It has been proposed that instead of neutralizing antibody activity, ADCC response was detectable as early as 3 weeks after SIVmac251 infection [9], [10], [11]. ADCC activity has been recognized as an increasingly important consideration in comprehensive evaluations of HIV vaccines in humans or non-human primate model [12], [13].

Natural killer (NK) cells, as effector cells, play a crucial role in the ADCC response through their FcγRIIIa (CD16). It has been reported that NK cell-mediated ADCC was severely compromised in chronic HIV infection compared with healthy subjects or HIV elite controllers [14]. However, very limited data on the ADCC function of NK cells in non-human primates are available, resulting in a less comprehensive evaluation of HIV vaccines in the non-human primate model. The Letvin group[15] has depleted the CD16^+^ NK cells *in vivo* in rhesus macaques during SIV infection and found no significant difference in the control of SIV replication between groups with or without NK cell depletion. Although this experiment strongly suggests that the direct killing function of CD16^+^ NK cells does not contribute to the control of the virus, it does not eliminate the possibility that ADCC activity of the CD16^+^ NK subset may defend against SIV, as there are few SIV-specific antibodies in the sera during the first two weeks after SIV infection [11]. We are more likely to see a positive contribution from CD16^+^ NK cells later in SIV infection when more antibodies are present.

At present, the methods for detecting ADCC activity in monkeys, such as the rapid and fluorometric antibody-dependent cellular cytotoxicity assay (RFADCC), used human peripheral blood mononuclear cells (PBMCs) as the effector cells [11], [16]. However, there remains a difference between humans and monkeys in the effector cell-mediated ADCC response. To better understand the mechanism of ADCC in the non-human primate model, it is necessary to study the function of NK cells in monkeys in addition to the role of antibodies.

It has been reported that the frequency of CD16^+^ CD56^−^ NK cells is significantly decreased in SIV-infected rhesus macaques [17], [18]. Thus, we postulated that the decline of FcγRIIIa (CD16) baseline expression on NK cells might affect their ADCC function in the infected macaques. The FcγRII(CD32) found on NK cells in humans [19] was also evaluated in macaque NK cells to determine whether it played a role in the ADCC response. A class of proteins called the matrix metalloproteases (MMPs) mediate the loss of CD16 on NK cells in humans [20], [21] and correlate with the impaired ADCC function of NK cells in HIV infection [14]. In non-human primate model in the study of NeuroAIDS, macaques infected with SIVmac239 that expressed high level of MMP-9 in microglia showed more rapid disease progression (encephalitic) compared with control macaques expressing low level of MMP-9 [22]. Here, we hypothesized MMPs might have a similar effect on the CD16 expression and ADCC function of macaque NK cells.

In this study, a sensitive assay was applied to measuring NK cell-mediated ADCC function in rhesus macaques. Furthermore, we explored the differences in ADCC function of NK cells in healthy versus infected macaques, and evaluated possible factors that may affect NK cell-mediated ADCC, including expression of CD16, CD32 and the MMPs.

## Materials and Methods

### Animals and infections

A total of 73 Chinese rhesus macaques with an average age of 4.5 years were used in this study. All animals were free of SIV, SRV/D, STLV-1 and HBV before any experimental procedures. Macaques were housed at the Institute of Laboratory Animal Science, Chinese Academy of Medical Science (ILAS, CAMS). 46 healthy uninfected macaques, 16 macaques infected intravenously with SIVmac251, 6 macaques infected intravenously with SHIV-clade B (SF162p3) and 5 macaques infected intravenously with SHIV-clade C (constructed with Chinese HIV-1strains) were analyzed. The mean duration of all infection was 341 days (range, 305–485 days). The median viral load of all infection was 2,514 viral RNA copies/ml plasma (range, 108–225,047 viral RNA copies/ml).

All study procedures on macaques were approved by the Institutional Animal Care and Use Committee (IACUC) of Institute of Laboratory Animal Science, Chinese Academy of Medical Sciences (approval number: IACUC-MC-07-6003). ILAS facilities used in this study are fully accredited by the Association for Assessment and Accreditation of Laboratory Animal Care International (AAALAS). This study was carried out in strict accordance with the recommendations in the Guide for the Care and Use of Laboratory Animals of the Institute of Laboratory Animal Science (est. 2006) and with the recommendations of the Weatherall report “The use of non-human primates in research”. Great efforts in procedures were made to minimize stress, improve housing conditions as well as measures of animal amelioration of suffering in all work. All procedures were performed under anesthesia using ketamine hydrochloride. Animals were closely monitored and observed for development of disease at least twice daily. If the animals are determined to be under stress or in discomfort, appropriate anesthetics and/or analgesics are administered as directed by the clinical veterinary staff.

### Cell processing

Rhesus macaque PBMCs were isolated from EDTA-treated venous blood by Ficoll Hypaque centrifugation (Sigma) within 6 h of collection. After cell counting, PBMCs were resuspended in R10 medium (RPMI-1640 medium supplemented with 10% FBS, 2 mmol L-glutamine, 100 U/ml penicillin, 100 µg/ml streptomycin) for next experiments.

### Phenotyping

Cell surface staining was performed using the following monoclonal antibodies: CD3 (V450 conjugate, Clone SP34.2, BD Biosciences), CD8 (APC-cy7 conjugate, Clone SK1,BD Biosciences), NKG2A (PE conjugate, Clone Z199, Beckman- coulter), CD16 (FITC conjugate, Clone 3G8, BD Biosciences), CD56 (PE-cy5 conjugate, Clone NCAM16.2, BD Biosciences), CD32 (APC, FITC conjugates, Clone FLI8.26, BD Biosciences), CD69 (ECD conjugate, Clone TP1.55.3, Beckman-coulter). All data were collected on BD FACS Aria and analyzed by FlowJo software (TreeStar Inc.).

### Preparation of target cells

A single cell line p815 (mouse leukemic cell line) [14] was cultured with p815-specific antibodies originated from rabbits (ACCURATE CHEMICAL & SCIENTIFIC CORPORATION) for 1 h at 37°C in 5% CO_2_ in a total volume of 300 µl of R10 medium. Then Ab-coated p815 cells were washed twice with ice-cold R10. Control targets were non-coated p815 cells.

### NK cell-mediated ADCC assay

Rhesus macaque PBMCs were used as effector cells and stimulated with R10 medium, uncoated p815 cells, Ab-coated p815 cells, phorbolmyristate acetate and Ionomycin (Sigma), respectively. CD107a (PE-cy5 conjugate, Clone H4A3, BD Biosciences), Golgi-Stop (BD Biosciences) and Brefeldin A (Sigma) were added to each well and all samples were incubated for 12 h at 37°C in 5% CO_2_. Following culture, all samples were washed and stained with CD3, CD8, NKG2A, CD16 and CD56. Cells were then permeabilized using Caltag Fix & Perm (Invitrogen) and intracellular cytokine staining was carried out for IFN-γ (Alexa700 conjugate, Clone B27, Invitrogen) and TNF-α (APC conjugate, Clone MAb11, BD Biosciences). After staining, cells were washed and fixed by 2% PFA. All data were acquired on BD FACS Aria and analyzed by FlowJo software.

### Fcγ RII blockade assay

Blocking antibodies FcγRII (10 µg/ml, R&D Systems) were added to PBMCs and cultured for 1 h at 37 °C. Either antibody-coated or uncoated p815 cells were added and cultured for 15 min or 12 h. The ADCC function of NK cells was evaluated at the two time points by flow cytometry using the NK cell-mediated ADCC assay as described above.

### MMP blockade assay

PBMCs were incubated with medium, Ab-coated or uncoated p815 cells in the presence or absence of MMP inhibitor GM6001 (Millipore) for 15 min or 12 h. The phenotypic changes of NK cells incubated with medium or MMP inhibitors (10 µg/ml) for 12 h were monitored by flow cytometry. The functional changes of NK cells incubated with the Ab-coated p815 cells or uncoated p815 cells were evaluated by flow cytometry at the time point of 15 min and 12 h for ADCC assay following treatment with or without MMP inhibitors (10 µg/ml). DMSO was added in every negative control group.

### Plasma concentration of MMP-9

The plasma levels of MMP-9 in macaques were determined by ELISA (enzyme-linked immunosorbent assay). Commercial ELISA kit (eBioscience) was used in the assay and the test process was in accordance with the manufacturer's instructions.

### Viral load quantification

Plasma viral RNA was determined in EDTA-treated venous blood using ABI PRISM 7700 Sequence Detector. On the basis of amplification of *gag*, the assay used a standard real-time reverse transcriptase polymerase chain reaction with a detection limit of 100 viral RNA copies per milliliter of plasma.

### Statistical analysis

All the statistical and graphic analyses were done using GraphPad Prism 5.0 software (GraphPad Software Inc.) and Sigma Plot 10.0 software (SPSS Inc.). Student's *t*-test was used in the comparisons of data from naive and infected animals when normality test passed. Non-normality data were analyzed by Mann-Whitney *U*-test. Spearman's rank correlation was used to analyze the correlation between variables. *P* values less than 0.05 were considered to be significant in the study.

## Results

### Method used for measuring NK cell-mediated ADCC

**Figure 1:**
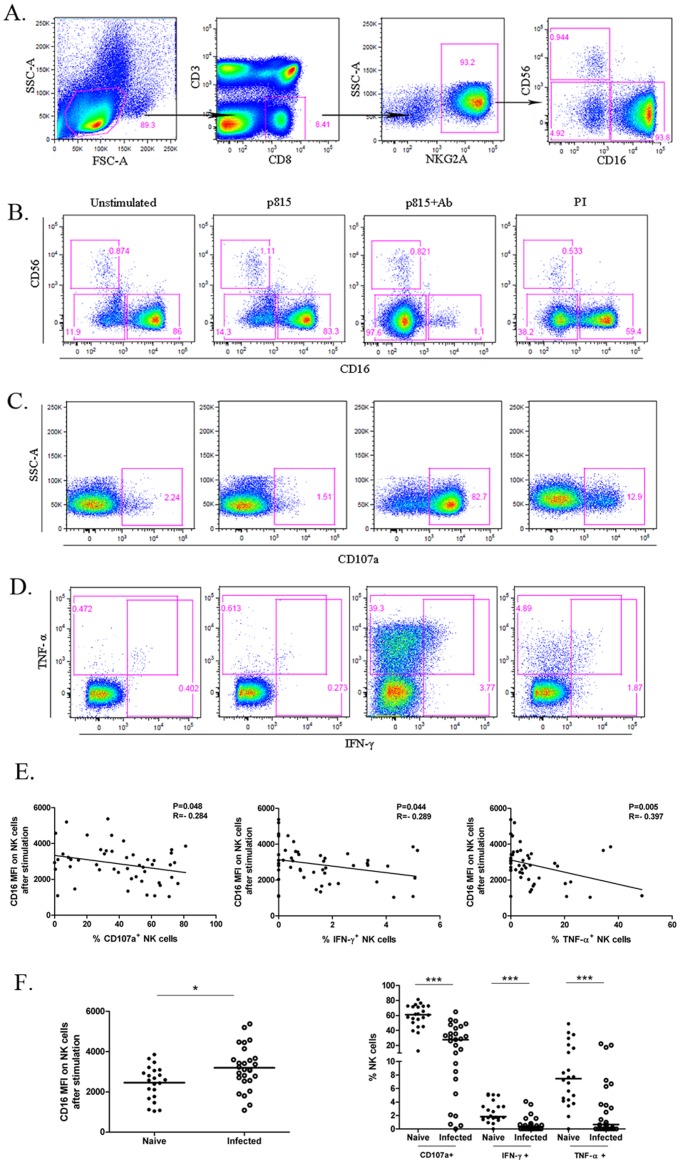
Definition of macaque NK cells and their response to Fc targets in the naive and SIV/SHIV infected macaques. (A) Macaque NK cells were defined as CD3^−^CD8^+^NKG2A^+^. 3 subsets were further divided by the expression of CD16 and CD56. Levels of CD16 expression on NK cells (B) as well as CD107a (C), IFN-γ and TNF-α (D) were measured. The flow plots represent the capacities of macaque NK cells to respond to medium alone, the mouse cell line p815, p815 cells coated with p815-specific Abs (Fc targets) and PMA+Ionomycin (from left to right ) respectively. (E) Correlation between CD16 MFI after stimuli by Fc targets and functional molecules are illustrated. Spearman Rank Order Correlation coefficients ‘R’ and corresponding ‘P values’ are indicated. (F) Chronic SIV/SHIV infection was associated with compromised NK cell-mediated responses to Fc targets in terms of the intensity of CD16 after stimulation (left; Horizontal bars indicate means for the normality data analyzed by Student's *t*-test ;*P<0.05 ) and the production of functional molecules (right; Horizontal bars indicate medians for the non-normality data analyzed by Mann-Whitney *U*-test; ***P<.001).

Recent research has proposed the use of NKG2A^+^, a major subset in CD3^−^ CD8^+^ cells, to identify macaque NK cells [18], [23], [24]. Therefore, in this study, macaque NK cells were identified as CD3^−^CD8α^+^NKG2A^+^ comprising three major subsets: CD16^+^CD56^−^(hereafter referred as CD16^+^ NK cells), CD56^+^CD16^−^, CD16^−^CD56^−^ (DN). Differing from NK cells in humans, macaque NK cells with little CD56 expression were dominated by the CD16^+^ subset (Figure 1A).

In order to exclusively detect the capacity of macaque NK cells to mediate ADCC, we chose a single cell line p815 coated with p815 specific antibodies as the target cells (Fc targets), which has been used in human NK cell-mediated ADCC assay [14]. FcγRIIIa (CD16) is a low-affinity receptor that only binds to clustered IgG displayed on the cell surface but not to monomeric IgG. Human studies have demonstrated that CD16 shedding occurs through matrix matalloproteases (MMPs) following NK cell stimulation [14]. In our study, stimulation with Ab-coated p815 cells led to an obvious decrease in the CD16^+^ population in macaque NK cells but not in the CD56^+^ subset (Figure 1B). Slighter change in the CD16^+^ NK subset was also observed under the circumstance of stimulation by phorbolmyristate acetate and Ionomycin (PI), but not in the presence of medium or uncoated p815 cells. These results strongly supported the conclusion that NK cells in macaques, analogous to human NK cells, downmodulated CD16 expression after activation, which was particularly significant in response to Fc targets.

**Figure 2:**
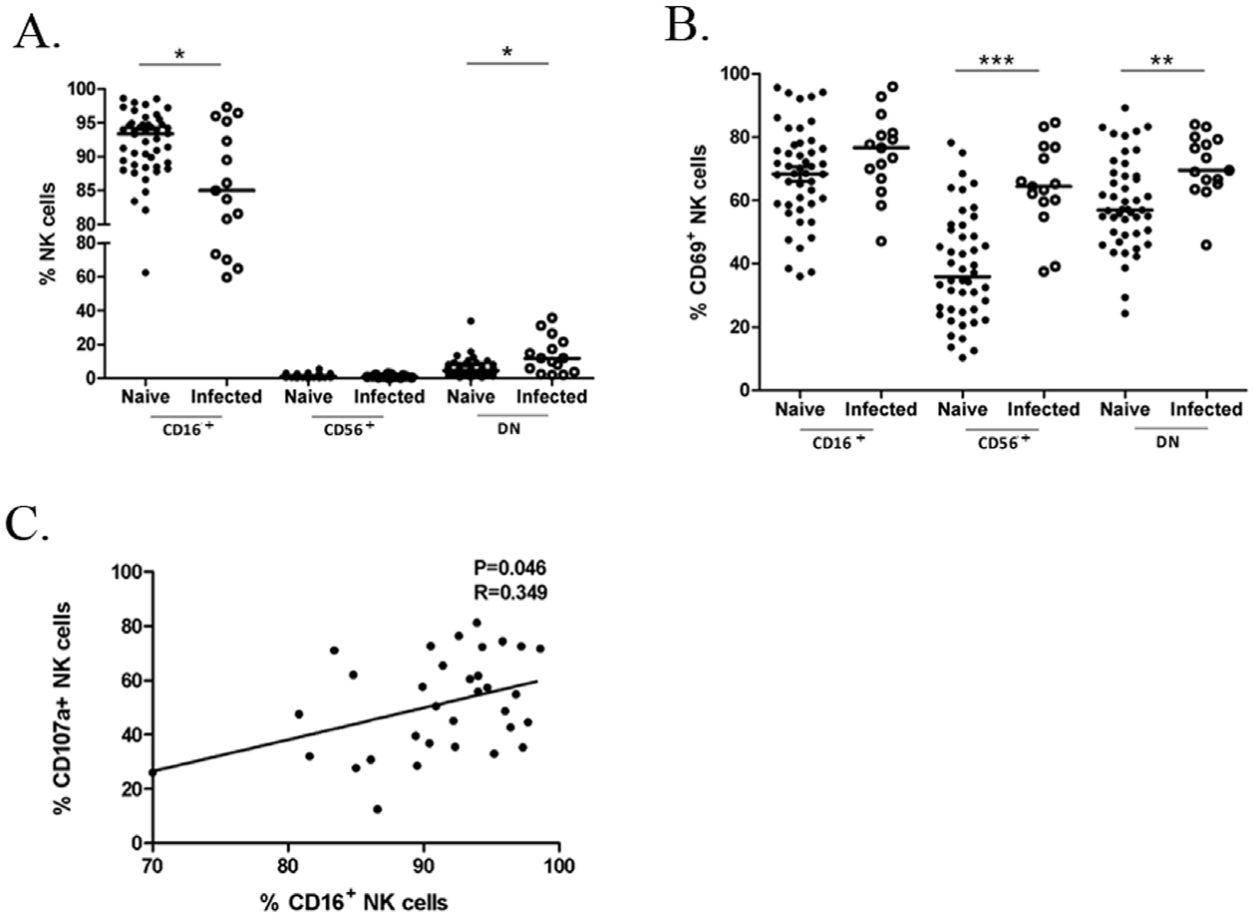
Phenotypic differences of NK-cell subsets in the naive and SIV/SHIV infected macaques. (A) CD16 (FcγRIII) expression is strongly downregulated in the chronically infected group. Horizontal bars indicate medians for the non-normality data analyzed by Mann-Whitney *U*-test; *P<.05. (B) Expression of CD69 increases in the infected group compared with the naive. Horizontal bars indicate means for the normality data analyzed by Student's *t*-test; **P<.01;***P<.001. (C) Correlation between the expression of CD16 and production of CD107a in NK cell-mediated ADCC is illustrated. Spearman Rank Order Correlation coefficients ‘R’ and corresponding ‘P values’ are indicated.

Expression of CD107a, a degranulation marker, can be used as an indicator of cellular cytotoxic activity [25]. After culturing with Ab-coated p815 cells, NK cells markedly increased expression of CD107a compared with those cultured in medium alone or uncoated p815 cells (Figure 1C). In addition, levels of IFN-γ and TNF-α secreted by NK cells were also significantly raised in response to Fc targets (Figure 1D). We found the trend correlating CD107a expression and cytokine release with CD16 loss after stimulation, raising the possibility of using the CD16 marker as indicator of ADCC function of NK cells in macaques. Hence, we focused on the relationship between the functional molecules and the baseline CD16 expression or CD16 expression after stimulation on NK cells from 49 macaques. As shown in Figure 2C, the expression of CD107a in ADCC response was significantly associated with CD16 expression on NK cell surface (*P* = 0.046, R = 0.349). Moreover, all of the three indicators, CD107a, IFN-γ and TNF-α, had negative but weak relationships with the CD16 MFI on NK cells after stimulation in the ADCC response (CD107a%: *P* = 0.048, R = −0.284 ; IFN-γ%: *P* = 0.044, R = −0.289; TNF-α%: *P* = 0.005, R = −0.397) (Figure 1E).Thus, the increased level of CD16 allowed NK cells to better respond to Fc targets and higher CD16 expression after stimulation might be an indication of defective capacity of NK cells to mediate ADCC.

### Compromised NK cell-mediated ADCC in chronic SIV/SHIV Infection

To observe phenotype changes in NK cells during SIV/SHIV infection, we assayed the distribution of the three NK subsets from 15 chronically SIV/SHIV infected and 46 healthy macaques. Significantly reduced expression of CD16 on infected macaque NK cells was observed in comparison with the naive group, accompanied by an increased proportion of DN subsets (*P*<0.05 for both) (Figure 2A). CD56^+^CD16^−^ subsets showed no difference between the two groups. Decline of CD16 expression was an indication of the inability of NK cells to mediate ADCC in the infected macaques.

CD69 is an activation marker expressed on NK cells and is up-regulated following stimulation [26]. In the study, a significantly higher level of CD69 expression on NK cells was observed in the infected cohort (*P*<0.05). In terms of the three NK-cell subsets, we found the CD69 expression was modestly upregulated in the CD16^+^ subset (*P* = 0.096) and notably upregulated in the CD56^+^CD16^−^ subsets (*P*<0.001) or DN subsets (*P*<0.01) compared with that from the naive cohort (Figure 2B). Increasing expression of CD69 suggested activation of NK cells after SIV/SHIV infection. However, NK cells from infected macaques had a reduced capacity in response to Fc targets, as noted by the significant higher level of CD16 expression after stimulation in terms of the mean fluorescence intensity (MFI) in the infected group than the naive group (*P*<0.05) (Figure 1F). Furthermore, we found that the median frequency of CD107a^+^ NK cells from normal macaques (median, 61.12%; range, 50.54%–72.29%) was over 2-fold higher than infected macaques (median, 27.78%; range, 7.68%–40.95%). Cytokine production in the NK cells from the infected group was also notably reduced (IFN-γ^+^ %:median, 0.35%; range, 0%–0.75%; TNF-α^+^ %:median, 0.66%; range, 0%–3.62%) compared with the naive group (IFN-γ^+^ %: median, 1.83%; range, 1.60%–3.32%; TNF-α^+^ %: median, 7.45%; range,4.31%–16.37%) (Figure 1F). Additionally, no significant difference was observed between the SIV and SHIV infected group in terms of either CD16 expression or the NK cells responding to Fc targets. These data confirm that SIV/SHIV infection had a significant impact on the capacity of NK cells to respond to target cells, muting their ADCC function from the aspects of both cytotoxicity and cytokine production in the chronic phase. In the study, we found no correlation between ADCC activity and CD4 T-cell counts or viral loads.

### Functional profile of NK cells activated in the ADCC assay

**Figure 3:**
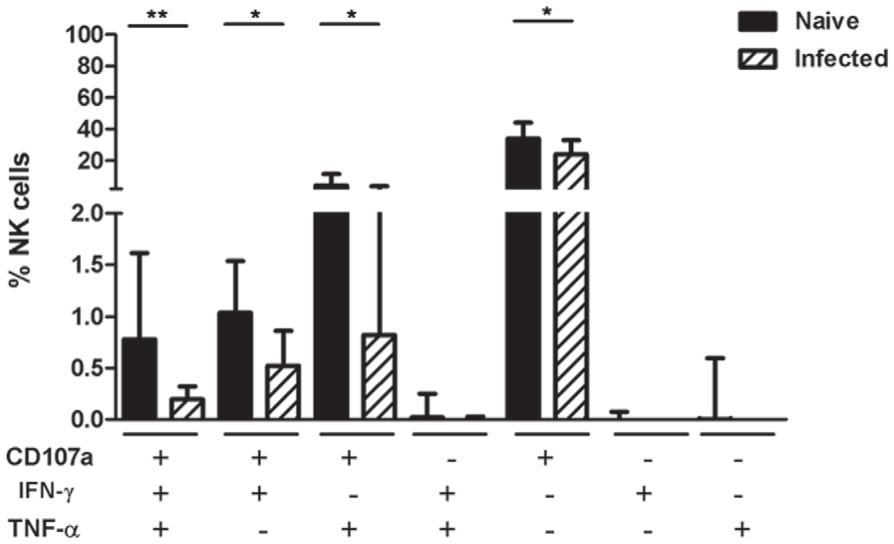
Functional profile of NK cells in naive and SIV/SHIV infected macaques. Total NK cells were divided into 7 subpopulations based on their production of CD107a, IFN-γ and TNF-α individually or in combination. The proportions of NK cells producing a given function were highly disparate between the 22 naive and 27 infected macaques, which are shown with medians and interquartile ranges in the graph. Mann-Whitney *U*-test; *P<.05;**P<.01.

As previous studies have indicated that polyfunctional cellular response, including CTL and NK-cell response, are associated with the control of HIV infection [27], [28], [29], we analyzed the functional repertoire of antibody-activated NK cells in the naive and infected macaques to see if there was a difference in polyfunctional cells between the two groups (Figure 3). Notably, the expression of CD107a overwhelmingly dominated the functional proportion both in naive and infected macaques, which suggested that degranulation was major response of macaque NK cells when exposed to Fc targets. The NK cells in naive macaques produced a triple-function composition at significantly higher frequency than the infected macaques (*P*<0.01). Dual-functional cells expressing CD107a and IFN-γ as well as CD107a and TNF-α showed higher percentage of NK cells in the normal group than the infected group (*P*<0.05 for both). There was also significant difference in mono-function of CD107a between the two groups (*P*<0.05). These data suggested that polyfunctional NK cells responding to Fc targets occurred more frequently in healthy macaques.

### A novel marker CD32 related to ADCC on macaque NK cells

**Figure 4:**
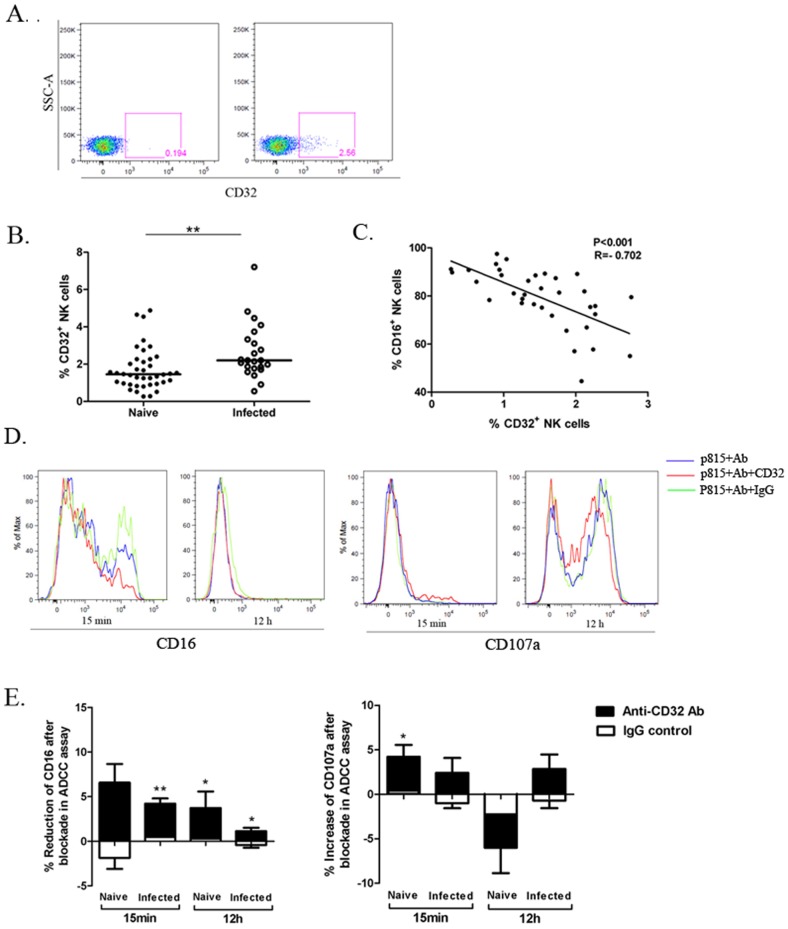
CD32 expression on the NK surface in the naive and SIV/SHIV infected rhesus and its impact on NK cell-mediated ADCC in macaques. (A) The flow plots represent the CD32 expression on macaque NK cells (right) with the isotype-negative control (left). (B)The comparison of CD32 expression on NK cells from the 40 naive macaques and 23 infected macaques was analyzed. Horizontal bars indicate medians. Mann-Whitney *U*-test; **P<.01. (C) Correlation between expression of CD16 and CD32 on macaque NK cells is illustrated. Spearman Rank Order Correlation coefficients ‘R’ and corresponding ‘P values’ are indicated. (D) Representative histogram overlays show the expression of CD16 and CD107a on a healthy macaque NK cells activated by Fc targets at 15 min and 12 h in the presence or absence of CD32 blocking antibodies. Irrelevant murine IgG was used as negative control in the blockade assay. (E) The bars represent the reduction of CD16 and increase of CD107a expression on NK cells from 12 healthy and 6 infected macaques in ADCC response after CD32 blockade at 15 min and 12 h. Irrelevant murine IgG was used as negative control. Data represent mean ± SEM. Student paired *t* test;*P<0.05; **P<.01.

In addition to CD16, NK cells in humans express another class of low-affinity receptors for IgG, called FcγRII(CD32), which like CD16, is able to bind to Fc region of IgG displayed on cells [19]. This raised the possibility that CD32 may also be expressed on NK cells from rhesus macaques. PBMCs from the 40 naive and 23 infected macaques were analyzed for expression of CD32 on macaque NK cells by flow cytometry. CD32 marker was observed on NK cells, albeit at low level (Figure 4A). The expression of CD32 on NK cells from the naive and SIV/SHIV infected groups was found to be significantly different (*P*<0.01) (Figure 4B). Furthermore, the expression of CD32 was negatively correlated with CD16 expression on macaque NK cells (*P*<0.001, R = −0.702; Figure 4C).

It has been demonstrated that one isoform of FcγRII, called FcγRIIB (CD32B), expressed on human NK cells results in inhibition of cell activation [19]. Therefore, the functional activity of CD32 on NK cells was tested to determine if they had an impact on the ADCC profile in macaques. Given the recent report on inhibition of early CD16 signal transduction of NK cell-mediated ADCC in HIV infection [30], we also want to know if CD32 expression could affect the early events in CD16 cross-linking on macaque NK cells responding to Fc targets. We measured the kinetics of NK cell-mediated ADCC in CD32 blockade study through changes of CD16 or CD107a expression in healthy macaques (Figure S1) and found the 15-min and 12-hour were the most representative time points for early and late events in ADCC response, respectively. Then, 12 healthy macaque NK cells (CD107+% in ADCC assay: median, 66.35%; range, 31.70%–90.40%) and 6 infected macaque NK cells that had severely impaired capacity in response to Fc targets (CD107+%: median, 11.72%; range, 0.5%–31.13%) were measured in the CD32 blockade assay. The percentage of CD16 was decreased in the CD32 blocking group compared with the unblocking group during the ADCC response (*P* = 0.063 for healthy group and *P* = 0.003 for infected group at 15 min; *P* = 0.016 for healthy group and *P* = 0.030 for infected group at 12 h) and was stronger at the early time point (Figures 4D and 4E), suggesting that the CD32 blockade had impact on early events triggered by CD16 cross-linking on macaque NK cells and might accelerate the ADCC response. Compared with the group in the absence of CD32 blocking antibodies, the percentage of CD107a^+^ NK cells in the blocking group tended to shift from increase at 15 min (*P* = 0.039 for healthy group and *P* = 0.221 for infected group) to decrease at 12 h (*P* = 0.061) among healthy macaques, but rose continuously among infected macaques with severely defective ADCC function at 12 h (*P* = 0.156). A modest difference was noticed between the naive and infected group in the effect of CD32 blockade at 12 h (*P* = 0.068).

### Restoration of ADCC dysfunction by MMP inhibitors

**Figure 5:**
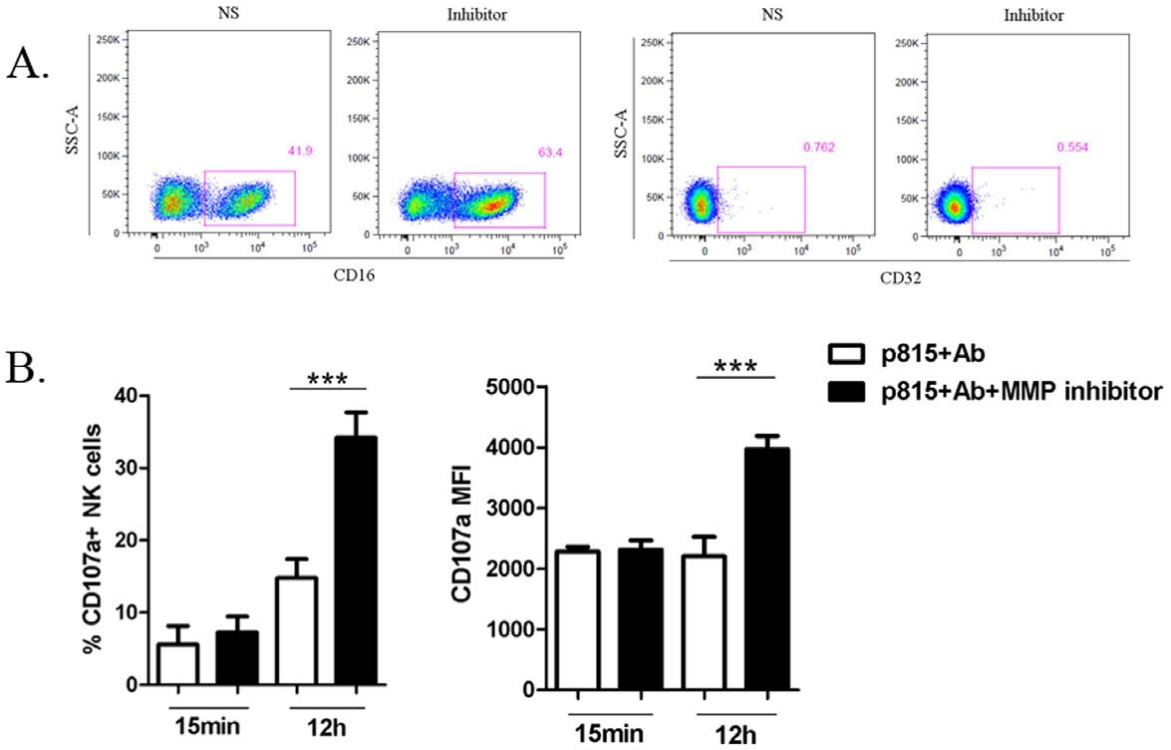
Restoration of NK cell-mediated ADCC by MMP inhibitors. (A) The flow plots depict changes of CD16 and CD32 expression on NK cells from one infected macaque in the presence or absence of MMP inhibitors at 12 h. (B) The bars in the graph represent changes in the capacity of NK cells from 14 infected macaques to exert cytotoxicity in ADCC response following treatment with MMP inhibitors at 15 min or 12 h. Data represent mean ± SEM. Student paired *t* test;***P<.001.

MMPs are known to be responsible for CD16 shedding after NK cell stimulation in humans [21]. To identify whether MMPs had any impact on phenotype of macaque NK cells, we measured expression of the two Fc receptors, CD16 and CD32, on NK cells from 12 infected macaques in the presence or absence of the MMP inhibitor GM6001. After culturing with MMP inhibitors for 12 h, CD16 expression on macaque NK cells increased significantly (*P*<0.001) (Figure 5A). No notable change in CD32 expression was observed in the blockade assay. The enhancement of the CD16 expression on macaque NK cells by MMP inhibitors implied that MMP blockade might restore the capacity of NK cells to mediate ADCC.

A previous study has demonstrated that MMP inhibitors can promote the magnitude of NK cell-mediated ADCC in humans [14]. Thus, we compared the cytotoxic capacity of 14 infected macaque NK cells in the inhibitor-treated and untreated groups by ADCC assay at 15 min and 12 h, which simulated the early and late events of ADCC activity respectively. After MMP blockade, degranulation of NK cells in response to Fc targets increased minimally at 15 min but elevated significantly at 12 h in terms of either percentage or intensity of CD107a expression (*P*<0.001 for both) (Figure 5B). These data suggested that MMP inhibitor treatment promoted the level of CD16 expression on macaque NK cells, allowing them to better respond to Fc targets in ADCC activity. We also detected the plasma levels of MMP-9 in 8 healthy and 12 infected macaques by ELISA and found a trend of increased MMP-9 in the infected macaques compared with healthy group, but the difference was not statistically significant (Figure S2).

## Discussion

The recent RV144 Thai trial has revealed a novel mechanism of immune protection that is related neither to neutralizing antibodies nor cytotoxic T lymphocytes but, instead, to non-neutralizing antibodies [13], offering researchers a new clue for eliciting protection against HIV infection. Under these circumstances, ADCC, which has been shown to be effective in vaccine trials in non-human primates and in the control of HIV infection in patients, becomes an important consideration. However, effector cells, as central performers of ADCC, are poorly studied in comparison with antibodies, especially in non-human primates. Based on our integrated understanding of the mechanism of ADCC and its role in the control of retrovirus-induced human disease, further attention to NK cells in the non-human primate model is warranted.

In this study, a sensitive ADCC measurement for macaque NK cells had been used based on flow cytometry assay. After stimulation with Ab-coated p815 cells, CD16 expression on NK cells was markedly downregulated, which was probably mediated through MMPs. Meanwhile, NK cells were triggered to degranulate and to secrete a large number of cytokines such as IFN-γ and TNF-α. We also discovered that the degranulation of NK cells in response to Fc targets closely correlated with the baseline of CD16 expression, indicating that the increased expression of CD16 enables NK cells to mediate ADCC more effectively.

Consistent with previous findings [24], the upregulation of CD69 on NK cells was observed in the infected cohort, indicating the activation of NK cells in defense against SIV/SHIV infection. CD69 expression has been reported to identify cells in a state of anergy post function as opposed to the cells which are ‘pre-activated’ and ready to function [26]. Thus, higher CD69 expression on NK cells could suggest the impaired capacity of NK cells to respond to Fc targets in the chronic-infected macaques. We found a contraction of baseline CD16 expression on NK cells in the chronic-infected cohort, which was a sign of defective NK cell-mediated ADCC. Not only the higher level of CD16 expression after Fc target stimulation but also the decreased production of CD107a and cytokines were observed in the NK cells from chronically infected macaques in ADCC assay, accompanied by the significant decline of polyfunctional NK cells. Thus, there was an overall compromised ADCC activity of NK cells with the association of the decreased baseline CD16 expression in the chronic SIV/SHIV infection. According to the hypothesis of ‘anergy post function’, these defects in NK cells as ADCC effectors in chronic group might indicate their high ADCC activity in the early SIV/SHIV infection *in vivo*.

Furthermore, we identified another marker FcγRII(CD32) that could also bind IgG like CD16 but correlated negatively with CD16 in terms of baseline expression on macaque NK cells. CD32 appeared to attenuate and/or delay the activation of macaque NK cells responding to Fc targets when NK cells were not completely activated in the case of early ADCC activity. Furthermore, at the late stage of ADCC, in contrast with the impact of CD32 blocking on the NK cells from the naive group, the blockade of CD32 resulted in a modest but not significant increase of cytotoxic capacity of NK cells in the infected macaques that had severely damaged NK cell-mediated ADCC. The obstruction of CD32 in the course of NK-cell activation raises the possibility that the significantly higher baseline CD32 expression on the infected macaque NK cells might account for their decline and/or delaying ADCC activity. The signal transduction of CD32 and its potential interplay with the signal transduction triggered by CD16 cross-linking on NK cells in rhesus macaques warrants further study, which might reveal the deeper mechanism of reduced capacity of NK cells in ADCC response. Additionally, it is possible that adding blocking antibodies against CD32 may affect first and foremost the non-NK cells with predominant CD32 expression in macaque PBMCs, such as monocytes and dendritic cells [31]. Thus, the impact of blocking CD32 on NK cell-mediated ADCC in this study could also be an indirect consequence.

Previous studies documented that MMPs play an important role in regulating NK-cell function though the enzymatic removal of some activating receptors, like CD16 [20], [21], while the use of MMP inhibitors restored the compromised NK cell-mediated ADCC in individuals with HIV-1 infection [14]. Here we found that blocking the activity of MMPs in rhesus macaques by MMP inhibitors resulted in an increase in CD16 expression, which suggests MMPs are probably responsible for the lost CD16 expression of NK cells in the infected macaques. In addition, MMP inhibitors could reconstitute NK cell-mediated ADCC, which was consistent with CD16 upregulation. The MMP inhibitors used in clinical trials for cancer or other disease [32], [33] are also considered as a rational therapeutic strategy for HIV infection [34]. Our study on MMPs from the aspect of NK cell-mediated ADCC in the non-human primate model supports this approach.

The method used for measuring NK cell-mediated ADCC could also be applied to evaluate ADCC function of other effector cells bearing FcγR. It has been reported that γδT cells can be activated to become cytotoxic effectors for ADCC through CD16 in humans [35], [36], [37]. Our group also evaluated γδT cell-mediated ADCC in rhesus macaques and found that macaque γδT cells that could be stimulated strongly by phosphoantigen isopentenyl pyrophosphate (IPP) had little response to Fc targets regardless of infection (unpublished data), implying that NK cells may be the main effector cells for ADCC in macaques.

In conclusion, our analyses of the factors that can influence NK cell-mediated ADCC, i.e., CD16, CD32 and MMPs in the study, represent novel data on ADCC from the perspective of effector cells in macaques and lay a foundation for future work on this field. It is possible that the capacity of macaque NK cells to mediate ADCC is determined by the interaction between these factors. In humans, NK cells from the HIV-infected individuals have defective early signal transduction events in ADCC [30] and MMPs are also responsible for the impaired capacity of NK cells to respond to Fc targets in HIV-1 infection [20], [21]. The compromised ADCC function of NK cells in the chronically infected macaques might be due to blockade of the early CD16 signal transduction in ADCC which possibly involves increasing CD32 expression, or be the result of the potentially elevated MMP expression in the chronically infected macaques with loss of CD16^+^ NK cells. The underlying mechanisms of NK cell-mediated ADCC in macaques should be addressed in future studies. As NK cell-mediated ADCC was assessed in the chronically infected macaques here, relevant studies on infected macaques with early SIV/SHIV infection may reveal new information and further benefit the preclinical vaccination studies in non-human primates.

## Supporting Information

Figure S1 Impact of CD32 expression on ADCC function of NK cells from healthy macaques. The bars represent the reduction of CD16 and increase of CD107a expression on NK cells from 7 healthy macaques in ADCC response after CD32 blockade at different time points. Irrelevant murine IgG was used as negative control at 15 min and 12 h. Data represent mean ± SEM.
(TIF)

Figure S2 Plasma concentration of MMP-9 in naive and infected macaques. The plasma levels of MMP-9 in 8 naive and 12 chronically infected macaques were determined by ELISA (enzyme-linked immunosorbent assay). Data represent mean ± SEM.
(TIF)
