# Supplementary figures and images for: Compromised NK Cell-Mediated Antibody-Dependent Cellular Cytotoxicity in Chronic SIV/SHIV Infection
Source: PLoS One. 2013 Feb 12;8(2):e56309. doi: 10.1371/journal.pone.0056309 (PMC3570461; doi:10.1371/journal.pone.0056309)

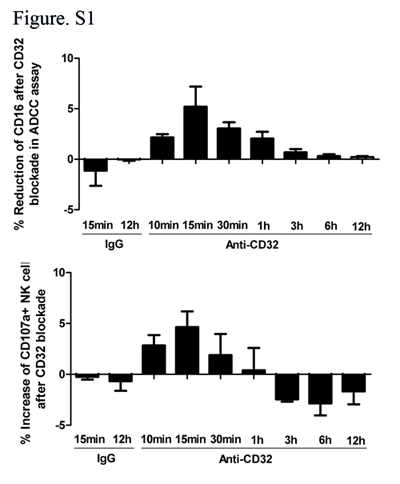

Supplement: Figure S1 — Impact of CD32 expression on ADCC function of NK cells from healthy macaques. The bars represent the reduction of CD16 and increase of CD107a expression on NK cells from 7 healthy macaques in ADCC response after CD32 blockade at different time points. Irrelevant murine IgG was used as negative control at 15 min and 12 h. Data represent mean ± SEM. (TIF) [file pone.0056309.s001.tif]

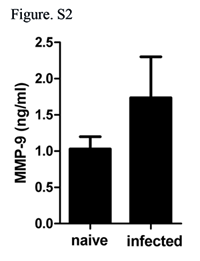

Supplement: Figure S2 — Plasma concentration of MMP-9 in naive and infected macaques. The plasma levels of MMP-9 in 8 naive and 12 chronically infected macaques were determined by ELISA (enzyme-linked immunosorbent assay). Data represent mean ± SEM. (TIF) [file pone.0056309.s002.tif]
